# The Use of Ethyl Chloride by the General Practitioner

**Published:** 1908-11-07

**Authors:** Lytton Maitland


### THE GENERAL PRACTITIONER'S COLUMN.

[Contributions to this Column are invited, and if accepted will be paid for.]

### THE USE OF ETHYL CHLORIDE BY THE GENERAL PRACTITIONER.

By LYTTON MAITLAND, M.B., B.S. (Lond.).

In spite of a disinclination still manifest among general practitioners and dentists to employ ethyl chloride more frequently, it may be confidently asserted that it is slowly but surely extending its sphere of usefulness from the hospital into private practice. In the past the reputation of ethyl chloride has suffered much from careless administrators, excessive dosage, and failure to realise its great rapidity of action. In general practice, its great convenience alone is a strong recommendation. Few general practitioners have a "gas" apparatus, and fewer still would think of carrying it to a patient's house, hence they fall back upon the use of chloroform even for short operations. It cannot be maintained that this practice is devoid of danger, especially in the oft-recurring operation for tonsils and adenoids. On the other hand, it must not be thought that the use of ethyl chloride is advocated in preference to gas when this is available, and will give sufficient length of anæsthesia. But for "tonsils and adenoids," opening abscesses thoroughly, removing toe-nails, scraping sinuses, and other small operations requiring one minute to a minute and a half, ethyl chloride is distinctly the most suitable anæsthetic. The practitioner saves the five to ten minutes required for the careful induction of chloroform anæsthesia, the patient is spared, in 90 per cent. of cases, all post-anæsthetic discomfort, and is, usually in half an hour, himself again.

One of the most striking features about ethyl chloride, besides its rapidity of action, is the important fact that the anæsthesia deepens after removal of the inhaler. Hence, in order to take full advantage of the time allowed, it is necessary that the surgeon should be ready as soon as the face-piece is applied.

One has sometimes had difficulty in persuading operators to begin owing to their unwillingness to believe that the patient is unconscious in about 45 seconds. In the case of children, it is not even necessary to wait for the total cessation of crying before allowing the operation to begin. This is especially true when the operation is not one on the mouth or throat. To keep the inhaler applied for longer than 50 or 60 seconds is a mistake, and will not have the effect of keeping the patient under any longer; in fact the reverse seems to be the result.

Before the stage of deep sleep, there is a pre-narcotic analgesic stage, in which an incision can be made painlessly; after the stage of complete unconsciousness there is another analgesic stage, which will afford more time to the operator. It is quite possible to amputate a finger or toe during one administration of ethyl chloride, though it will generally be necessary for an assistant to hold the limb steady, and one may have to defer the insertion of stitches until the patient has recovered his senses. On many occasions the writer has had to anæsthetise and operate single-handed—a feat quite practicable and followed by most satisfactory results, though naturally not a procedure of choice.

For adults a dose of 5 c.c. is the rule, and this should never be exceeded; if a slightly longer anæsthesia be required, a second dose of 3 c.c. will be ample to give the operator another minute, that is to say, a total of 2 to 2½ minutes. If the operation will take longer than this it is better to follow up the first dose of ethyl chloride by the exhibition of chloroform. Owing to the frequent occurrence of trismus it should be an invariable rule to insert, between the patient's teeth, a dental prop. This will allow a gag to be readily inserted for tonsillotomy directly the face-piece is removed and avoids the necessity of starting with the gag in the mouth—a procedure which not only frightens the child, but allows too much air to mix with the gas by preventing the accurate apposition of the face-piece.

Ethyl chloride will often be found to fail with young and vigorous alcoholics, such as sea-faring men, on account of the shortness of the stage of deep sleep and the violent muscular contractions, which prevent the pre-narcotic stage from being made use of by the surgeon. For children a dose of 3 c.c. should be given, and it is surprising how well this suffices; anæsthesia should be obtained in 20 to 30 seconds. The after-effects are wonderfully slight, and in a quarter of an hour the child has completely recovered. Vomiting is exceedingly rare if this dose is not exceeded.

The matter of preparation is as important as in the case of chloroform anæsthesia, and here the general practitioner usually has the advantage over the hospital out-patient surgeon. When one thinks of the enormous number of times ethyl chloride is administered in hospitals without untoward results, one may be excused in thinking that the general practitioner, whose patients are usually better prepared than the ordinary out-patient, might very well avail himself more often of this most convenient anæsthetic.

